# Angioscopic observation of venous endothelium after transvenous lead extraction with excimer laser sheath: Initial experience

**DOI:** 10.1016/j.hrcr.2022.07.020

**Published:** 2022-08-06

**Authors:** Satoshi Nakawatase, Hitoshi Minamiguchi, Atsushi Hirayama, Yoshiharu Higuchi

**Affiliations:** Department of Cardiology, Osaka Police Hospital, Osaka, Japan

**Keywords:** Nonobstructive angioscopy, Device infection, Transvenous lead extraction, Excimer laser sheath, Vascular injury

**Key Teaching Points**

• Cardiovascular implantable electronic device (CIED) removal is indispensable for CIED pocket infection; therefore, transvenous lead extraction (TLE) with excimer laser sheaths was performed in this case.
• Vascular injury is one of the most serious complications during TLE.
• We directly confirmed the venous endothelium after TLE and recognized the risk of vascular injury after TLE with excimer laser sheaths. These angioscopic findings might reveal the risk of vascular injury from excessive laser irradiation to strong adhesion tissue in the same region.

## Introduction

Cardiovascular implantable electronic device (CIED) removal is indispensable for treatment of CIED infection.^1^ Transvenous lead extraction (TLE) with excimer laser sheaths is performed in a case with CIED infection; however, it is unclear what venous endothelium looks like after TLE with excimer laser sheaths.

## Case report

A 57-year-old man with dilated cardiomyopathy was admitted to our hospital owing to need for TLE for pocket infection of his cardiac resynchronization therapy defibrillator. He underwent cardiac resynchronization therapy defibrillator implantation 4 years prior and had catheter ablation therapies performed 4 times for atrial fibrillation and atrial tachycardia.

**Figure 1:**
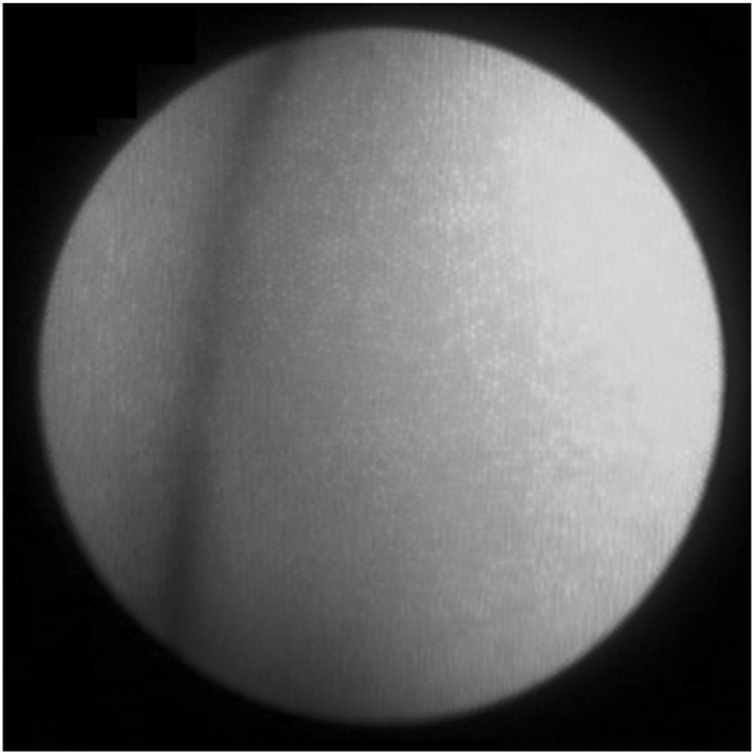
Before transvenous lead extraction, there was smooth and white normal venous endothelium.

**Figure 2:**
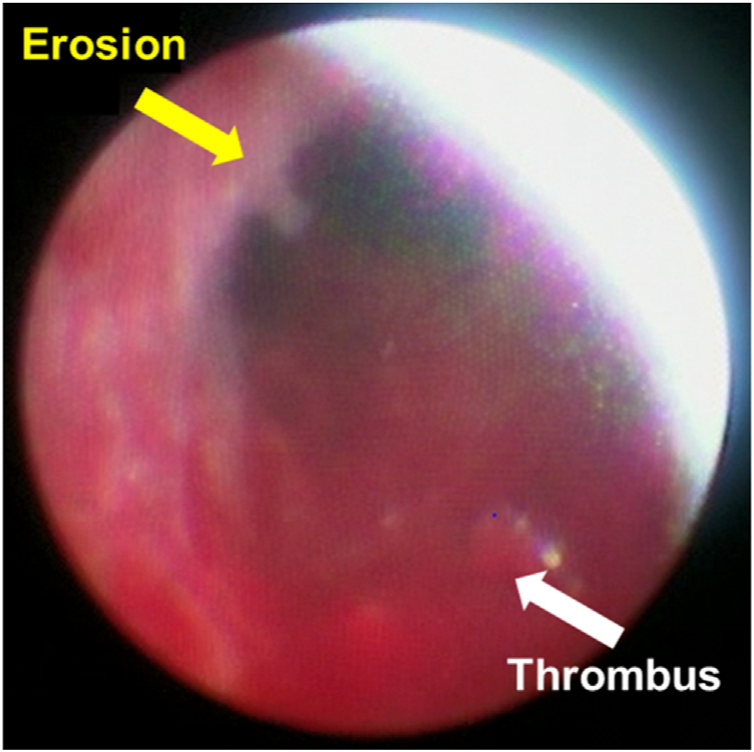
After transvenous lead extraction with the excimer laser sheaths, venous endothelium had erosions (*yellow line*) and adhesions of thrombi (*white line*).

We performed TLE under general anesthesia. We peeled off adhesions with all leads from axillary vein to right atrium using 12F and 14F excimer laser sheaths (Spectranetics, Colorado Springs, CO) and successfully extracted all leads, which were covered with adhesive tissue. Before and after using the excimer laser sheaths, we examined the condition of the venous endothelium in between the innominate vein and above the superior vena cava by nonobstructive angioscopy (Visible; Fiber Tech, Tokyo, Japan) through a femoral vein approach. Before use of the excimer laser sheaths, normal venous endothelium was smooth and white (Figure 1 and Supplemental Video 1); however, after use of the excimer laser sheaths, venous endothelium showed signs of erosion and adhesion of thrombus (Figure 2 and Supplemental Video 2).

After appropriate antibiotic administration and wound care, his wound completely healed and he was discharged from our hospital.

## Discussion

From these angioscopic findings, we directly recognized the risk of vascular injury after TLE with excimer laser sheaths. Vascular injury is one of the most serious complications during TLE procedures.^1^ These angioscopic findings might reveal the risk of vascular injury from excessive laser irradiation to strong adhesion tissue in the same region.

## AppendixSupplementary data

Movie legend Angioscopic observation was performed between in innominate vein and above the suviewperior vena cava. Before transvenous lead extraction, there was smooth and white normal venous endothelium (Video 1), however, after transvenous lead extraction with the excimer laser sheaths, venous endothelium had erosions and adhesions of thrombi (Video 2).
